# Targeting PPAR-γ to design and synthesize antidiabetic thiazolidines

**DOI:** 10.17179/excli2018-1325

**Published:** 2018-06-27

**Authors:** Ramesh L. Sawant, Jyoti B. Wadekar, Santosh B. Kharat, Hitakshi S. Makasare

**Affiliations:** 1 Department of Pharmaceutical Chemistry and PG studies, Dr. Vithalrao Vikhe Patil Foundation's College of Pharmacy, Savitribai Phule Pune University, Ahmednagar- 414111, India; 2 Department of Pharmacognosy, Dr. Vithalrao Vikhe Patil Foundation's College of Pharmacy, Savitribai Phule Pune University, Ahmednagar- 414111, India; 3 Department of Pharmaceutical Chemistry, VIVA Institute of Pharmacy, University of Mumbai, Palghar- 401303, India

**Keywords:** alloxan, antidiabetic, docking, PPAR-gamma, thiazolidine

## Abstract

A series of thiazolidine derivatives were designed by docking into PPAR-γ active site. The structure of target was obtained from the protein data bank (PDB ID P37231). A library of 200 molecules was prepared on random basis. Molecular docking studies were performed using VLife MDS 4.3 software. After molecular docking studies, the 4-substituted-6-methyl-2-thioxo-1,2,3,4-tetrahydropyrimidine-5-carboxylic acid N-[4-(2,4-dioxo-thiazolidin-5-ylidenemethyl)-phenyl]-hydrazides (**4a-4h**) were selected for synthesis. The progress of reaction and purity of the synthesized compounds were monitored by TLC and melting point. Structures of title compounds were confirmed by elemental analysis, IR, ^1^H NMR and mass spectral data. The antidiabetic activity of title compounds was performed using the Wistar rats by alloxan-induced method. The compounds have shown antidiabetic activity comparable with the standard drug pioglitazone. These findings suggest that potent antidiabetics can be generated by substituting nonpolar, electron withdrawing substituents at the fourth position of pyrimidine skeleton and hydrogen bond acceptor at the nitrogen of the thiazolidine nucleus, to inhibit peroxisome proliferator-activated receptor-γ.

## Introduction

Diabetes mellitus refers to a group of disorders that effects the body's ability to produce or respond to the hormone insulin, realizing abnormal metabolism characterized by hyperglycemia (Malecki and Klupa, 2005[7]). Globally, diabetes has shadowed the spread of modern lifestyle associated with an overweight and sedentary population (Moller, 2001[8]). The chronic metabolic disorder disturbs 150 million people and may rise to 300 million by 2025 worldwide (Kothari et al., 2005[6]). Diabetic patients suffer from cardiovascular, eye, kidney, and nerve damage which lead to premature handicap and death (Wright et al., 2006[16]).

Peroxisome proliferator-activated receptor (PPAR) belongs to the superfamily of a phylogenetically related protein named as nuclear hormone factor. PPAR-γ has a place with the group of PPAR which assume a basic part to regulate energy storage and its expression is in endothelial cells (Tyagi et al., 2011[15]). PPAR-γ activates some genes in tissues that results in an increase in glucose and lipid uptake, decreases free fatty acid concentration, and subsequently decreases the insulin resistance (Dumasia et al., 2005[3]). Thiazolidinediones (TZDs) are PPAR-γ ligands having an important structural domain for the design and synthesis of new drugs required to treat type 2 diabetes (Thangavel et al., 2017[14]). Thiazolidinedione and its derivatives possess diverse nonhypoglycemic effects, for example, against obesity (Bhattarai et al., 2010[1]), anti-atherosclerotic (Papanas and Maltezos, 2009[9]), antioxidant (Jeong et al., 2004[5]), anti-inflammatory (Shantharam and Chandy, 2011[12]), and anti-cancer (Patil et al., 2010[10]).

Molecular docking is an effective tool for investigating ligand-receptor interactions and for virtual screening, which plays a key role in rational drug design (Sawant et al., 2012[11]). Here, we describe the design, synthesis, and screening of a new series of 2,4-thiazolidine derivatives using molecular docking studies with the help of VLife MDS 4.3 software. Our efforts are dedicated to obtaining pyrimidine containing thiazolidine analogues having the affinity for PPAR-γ receptor as antidiabetics.

## Materials and Methods

### Molecular modelling

Docking studies were performed on VLife MDS 4.3 using grid-based docking method. The structure of the receptor PPAR-γ was retrieved from protein data bank (PDB ID-P37231). The receptor was opened in MDS sheet and saved as .mol2 format by removing the water molecule and this enzyme structure was used further for docking purposes. All the chemical structures were drawn in Chem Draw Professional version 15.1. Library of novel 200 analogues was prepared on the random basis. The protein-ligand complex was constructed and the active site of the enzyme was defined to include residues within a 10.0 Å radius to any of the inhibitor atoms. Batch docking was performed to generate dock score and interactions based on which eight different thiazolidinedione analogues were selected for synthesis. 

### Chemistry

**Figure 1:**
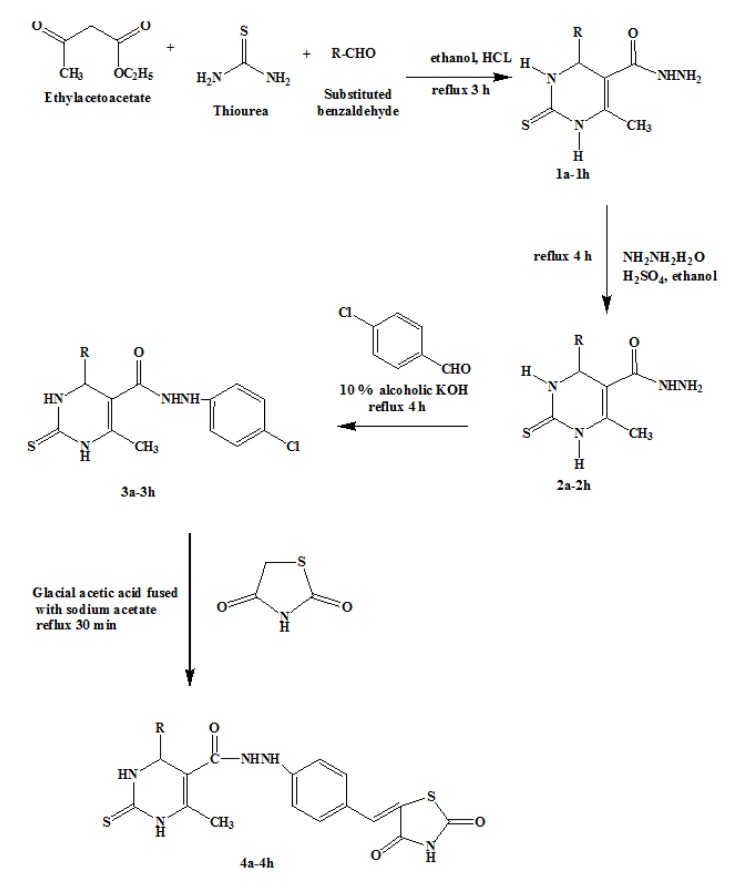
Synthesis of 2, 4-thiazolidine derivatives 4a-4h

Eight different 2,4-thiazolidine derivatives **(4a-4h)** were synthesized following Figure 1(Fig. 1). The melting point of the synthesized compounds was determined using Veego electronic (VMP-D) apparatus in an open capillary tube. The IR spectra were recorded on Shimadzu FTIR 8400S spectrophotometer and expressed in wave numbers (cm^-1^), using 1 % potassium bromide discs. The ^1^H NMR spectra were obtained on BRUKER AVANCEV II 400 NMR spectrometer using DMSO as solvent and TMS as an internal standard. Chemical shifts (δ) are given in ppm. The mass spectra were recorded to know the M+1 peak on LC-MS. The purity of the synthesized compounds was checked by silica gel G plate using chloroform: methanol (9:1) as the mobile phase.

#### General procedure for the synthesis of 6-methyl-4-phenyl-2-thioxo-1,2,3,4-tetrahydro-pyrimidine-5-carboxylic acid ethyl esters (1a-1h)

Thiourea (0.5 mol), ethyl acetoacetate (0.75 mol), and substituted aromatic aldehyde (0.5 mol) were mixed in 25 mL of ethanol. A catalytic amount of concentrated hydrochloric acid (5 drops) was added to the mixture and the mixture was refluxed for 3 h. On cooling, a solid separated was filtered and recrystallized from ethanol.

#### General procedure for the synthesis of 6-methyl-2-thioxo-4-phenyl-1,2,3,4-tetrahydro-pyrimidine-5-carboxylic acid hydrazide (2a-2h)

To the mixture of 0.1 mol of compound **1a-1h **in 20 mL ethanol, 0.1 mol of hydrazine hydrate and a catalytic amount of concentrated sulphuric acid was added and the mixture was refluxed for 4 h. On cooling, a solid separated was filtered and recrystallized from ethanol.

#### General procedure for the synthesis of 6-methyl-4-phenyl-2-thioxo-1,2,3,4-tetrahydro-pyrimidine-5-carboxylic acid N-(4-formyl-phenyl)-hydrazide (3a-3h)

To the mixture of 0.1 mol of compound **2a-2h **in 15 mL of 10 % alcoholic potassium hydroxide, 0.1 mol of *para*-chlorobenzaldehyde was added and refluxed for 4 h. On cooling, a solid separated was filtered and recrystallized from chloroform:ether (1:1).

#### General procedure for the synthesis of thiazolidinedione 

To 0.6 mol of chloroacetic acid in 60 mL of water, 0.6 mol of thiourea was added. The reaction mixture was stirred for 15 min and refluxed for 4 h. On cooling, a solid separated was filtered and recrystallized from ethanol.

#### General procedure for synthesis of 4-substituted-6-methyl-2-thioxo-1,2,3,4-tetrahydropyrimidine-5-carboxylic acid N-[4-(2,4-dioxo-thiazolidin-5-ylidenemethyl)-phenyl]-hydrazides (4a-4h)

To 0.25 mol of compound **3a-3h **in 50 mL of hot glacial acetic acid, 0.25 mol of thiazolidinedione, and 1.8 g of fused sodium acetate were added. The reaction mixture was refluxed under stirring for 1 h. The mixture was poured into 500 mL water and solid separated was filtered and washed with water, alcohol, and ether. The compounds were recrystallized from glacial acetic acid.

#### 6-Methyl-4-phenyl-2-thioxo-1,2,3,4-tetrahydropyrimidine-5-carboxylic acid N'-[4-(2,4-dioxo-thiazolidin-5-ylidenemethyl)-phenyl]-hydrazide (4a)

Melting point: 200-202°C, Yield: 65.82 % 

IR (KBR, cm^-1^): 3429.43 (NH stretch), 1651.01 (C=O), 1367 (C-N); ^1^H NMR (DMSO, 400 MHz, δ ppm): 1.71 (s, 3H, methyl), 2.0 (s, 2H, amine), 4.0 (s, 1H, Ar-NH), 4.59 (s, 1H, methine), 6.61-7.22 (m, 9H, CH), 7.57 (s, 1H, ethylene), 8.0 (s, 1H, sec amide), 10 (s, 1H, imid); MS: m/z (M+1): 465.12.

#### 4-(2-Chloro-phenyl)-6-methyl-2-thioxo-1,2,3,4-tetrahydropyrimidine-5-carboxylic acid N'-[4-(2,4-dioxo-thiazolidin-5-ylidenemethyl)-phenyl]-hydrazide (4b)

Melting point: 210-212°C, Yield: 67.58 %

IR (KBR, cm^-1^): 3429.43 (NH stretch), 1651.20 (C=O), 768 (C-Cl stretch); ^1^H NMR (DMSO, 400 MHz, δ ppm): 1.71 (s, 3H, methyl), 2.0 (s, 2H, amine), 4.0 (s, 1H, Ar-NH), 4.59 (S, 1H, methine), 6.61-7.22 (m, 8H, CH), 7.57 (s, 1H, ethylene), 8.0 (s, 1H, sec amide), 10 (s, 1H, imid); MS: m/z (M+1): 499.9.

#### 4-(4-Chloro-phenyl)-6-methyl-2-thioxo-1,2,3,4-tetrahydropyrimidine-5-carboxylic acid N'-[4-(2,4-dioxo-thiazolidin-5-ylidenemethyl)-phenyl]-hydrazide (4c)

Melting point: 225-228°C, Yield: 62.55 %

IR (KBR, cm^-1^): 3323.25 (NH stretch), 1664.57 (C=O), 766 (C-Cl stretch); ^1^H NMR (DMSO, 400 MHz, δ ppm): 1.71 (s, 3H, methyl), 2.0 (s, 2H, amine), 4.0 (s, 1H, Ar-NH), 4.59 (S, 1H, methine), 6.61-7.22 (m, 8H, aromatic CH), 7.57 (s, 1H, ethylene), 8.0 (s, 1H, sec amide), 10 (s, 1H, imid); MS: m/z (M+1): 499.9.

#### 4-(2-Hydroxy-phenyl)-6-methyl-2-thioxo-1,2,3,4-tetrahydropyrimidine-5-carboxylic acid N'-[4-(2,4-dioxo-thiazolidin-5-ylidenemethyl)-phenyl]-hydrazide (4d)

Melting point: 229-231°C, Yield: 60.59 % 

IR (KBR, cm^-1^): 3429.43 (NH stretch), 1651.01 (C=O), 1367 (C-N), 3245 (C-OH); ^1^H NMR (DMSO, 400 MHz, δ ppm): 1.71 (s, 3H, methyl), 2.0 (s, 2H, Ar-NH), 4.0 (s, 1H, Ar-NH), 4.59 (s, 1H, methine), 5.0 (s, 1H, Ar C-OH) 6.61-7.22 (m, 8H, aromatic CH), 7.15 (s, 1H, ethylene), 8.0 (s, 1H, sec amide), 10 (s, 1H, imid); MS: m/z (M+1): 481.5.

#### 4-(4-Methoxy-phenyl)-6-methyl-2-thioxo-1,2,3,4-tetrahydropyrimidine-5-carboxylic acid N'-[4-(2,4-dioxo-thiazolidin-5-ylidenemethyl)-phenyl]-hydrazide (4e)

Melting point: 263-265°C, Yield: 63.81 % 

IR (KBR, cm^-1^): 3420.13 (NH stretch), 1652.05 (C=O), 1367 (C-N), 2820 (O-CH_3_); ^1^H NMR (DMSO, 400 MHz, δ ppm): 1.57 (s, 3H, methyl), 2.0 (s, 2H, Ar-NH), 3.73 (s, 3H, methyl), 4.0 (s, 1H, amine), 4.59 (s, 1H, methane), 6.61-7.22 (m, 8H, aromatic CH), 7.15 (s, 1H, ethylene), 8.0 (s, 1H, sec amide), 10.0 (s, 1H, imid); MS: m/z (M+1): 495.57.

#### 6-Methyl-4-(4-nitro-phenyl)-2-thioxo-1,2,3,4-tetrahydropyrimidine-5-carboxylic acid N'-[4-(2,4-dioxothiazolidin-5-ylidenemethyl)-phenyl]-hydrazide (4f)

Melting point: 282-284°C, Yield: 61.80 % 

IR (KBR, cm^-1^): 3420.12 (NH stretch), 1650.01 (C=O), 1367 (C-N); ^1^H NMR (DMSO, 400 MHz, δ ppm): 11.10 (s, 3H, methyl), 2.0 (s, 2H, amine), 4.0 (s, 1H, Ar-NH), 4.59 (s, 1H, methine), 6.61-8.14 (m, 9H, aromatic CH), 7.15 (s, 1H, ethylene), 8.0 (s, 1H, sec amide), 10.0 (s, 1H, imid); MS: m/z (M+1): 510.1.

#### 6-Methyl-4-(2-nitro-phenyl)-2-thioxo-1,2,3,4-tetrahydropyrimidine-5-carboxylic acid N'-[4-(2,4-dioxothiazolidin-5-ylidenemethyl)-phenyl]-hydrazide (4g)

Melting point: 286-288°C, Yield: 62.56 %

IR (KBR, cm^-1^): 3429.43 (NH stretch), 1651.01 (C=O), 1367 (C-N); ^1^H NMR (DMSO, 400 MHz, δ ppm): 1.71 (s, 3H, methyl), 2.0 (s, 2H, amine), 4.0 (s, 1H, Ar-NH), 4.59 (s, 1H, methine), 6.61-8.07 (m, 8H, aromatic CH), 7.15 (s, 1H, ethylene), 8.0 (s, 1H, sec amide), 10.0 (s, 1H, imid); MS: m/z (M+1): 510.

#### 4-(3-Chloro-phenyl)-6-methyl-2-thioxo-1,2,3,4-tetrahydropyrimidine-5-carboxylic acid N'-[4-(2,4-dioxothiazolidin-5-ylidenemethyl)-phenyl]-hydrazide (4h)

Melting point: 237-239°C, Yield: 57.56 %

IR (KBR, cm^-1^): 3430.30 (NH stretch), 1651.01 (C=O), 1367 (C-N); ^1^H NMR (DMSO, 400 MHz, δ ppm): 1.71 (s, 3H, methyl), 2.0 (s, 2H, amine), 4.0 (s, 1H, amine), 4.59 (s, 1H, methine) 6.61-7.22 (m, 8H, aromatic CH), 7.15 (s, 1H, ethylene), 8.0 (s, 1H, sec amide), 10.0 (s, 1H, imid); MS: m/z (M+1): 499.

### Pharmacological screening

#### Antidiabetic activity

Wistar rats of either sex weighing between 150-200 g were utilized for the study. The animals were housed under controlled conditions with standard pellet diet and water ad libitum. The ethical clearance was obtained from the Institutional Animal Ethical Committee (Registration No.: 1670/PO/ReBiBt/S/12/CPCSEA, dated 02/05/2013). Diabetes was induced in the rats by intraperitoneal injection of alloxan monohydrate (S.D. Fine Chemicals, Mumbai) in the ice cold citrate buffer, pH 4.5 at a dose of 120 mg/kg. The diabetic state was confirmed 48 h after alloxan injection by the loss of body weight and hyperglycemia. The rats with blood glucose level 200-350 mg/dl were selected for the study. 

The rats were divided into three groups, each carrying six animals. Group I served as a diabetic control and received 0.3 % CMC, orally and alloxan. Group II served as a positive control and received a standard drug pioglitazone (0.04 g/kg). Group III comprised of 8 subgroups for 8 title compounds to be tested. Group IV served as the normal untreated group. The treatment was continued for 8 d by administering the test compounds in 0.3 % CMC orally. On the ninth day, blood samples were collected by the tail tip cut method under mild ether anesthesia. Serum was separated by centrifuging the samples at 6000 rpm for 20 min and stored in the refrigerator until analyzed. The serum analysis was performed for blood glucose level, cholesterol, triglycerides, and low-density lipoprotein (LDL) following the kit manual. The quantitative measurements were made on six animals in each group and the value of biochemical estimations are expressed as mean ± SE. The data obtained were subjected to one-way ANOVA followed by Tukey test.

## Results and Discussion

### Docking studies

**Figure 2:**
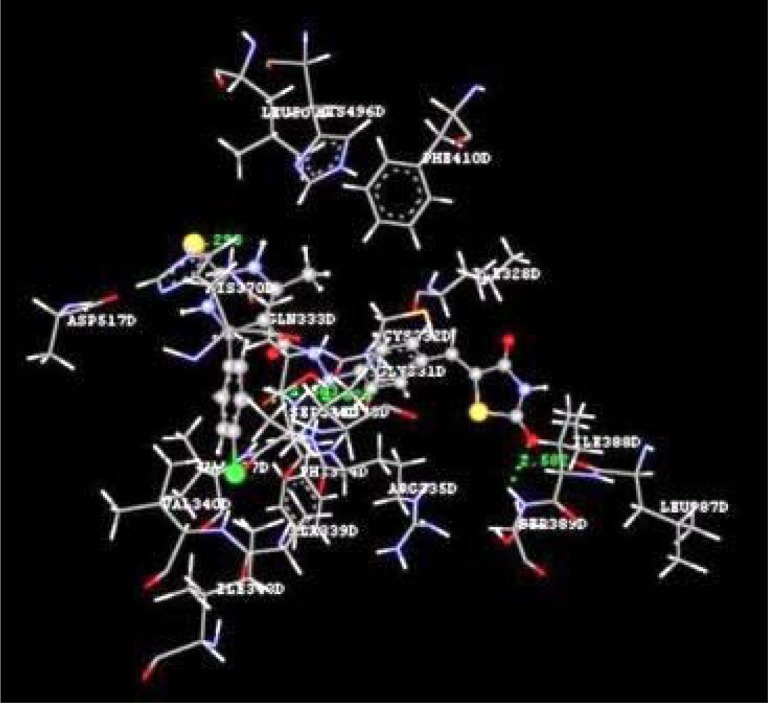
Ligand receptor interaction diagram showing hydrogen bond interactions of compound 4b at the binding site of PPAR-γ

**Figure 3:**
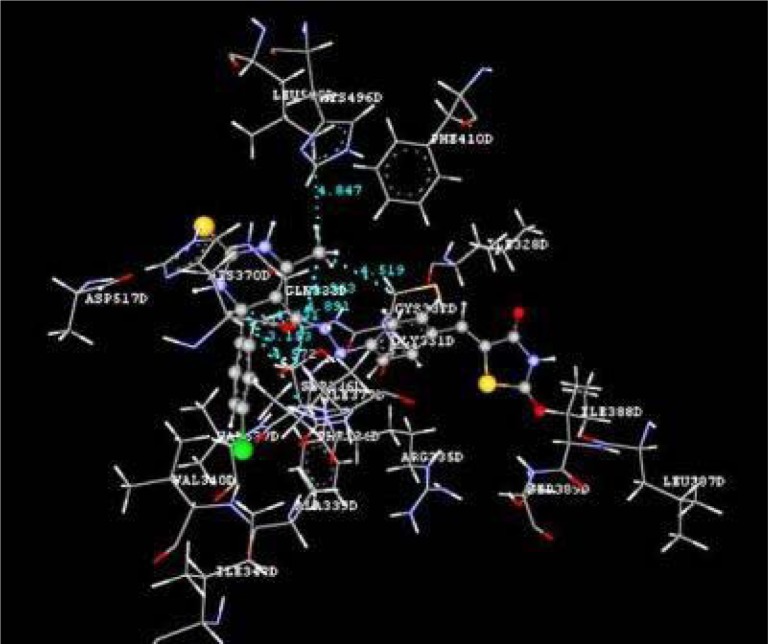
Ligand receptor interaction diagram showing hydrophobic interactions of compound 4b at the binding site of PPAR-γ

**Figure 4:**
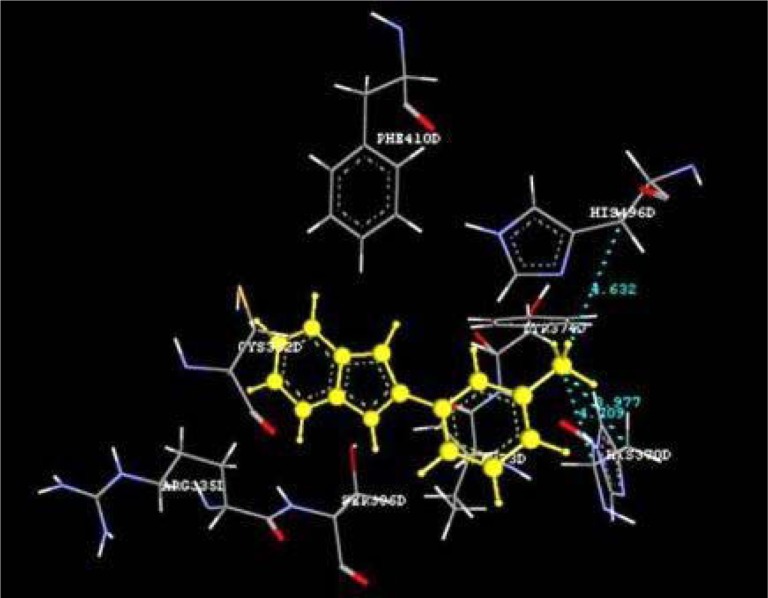
Ligand receptor interaction of standard drug pioglitazone at the binding site of PPAR-γ

Docking studies of the title compounds with PPAR-γ yielded docking score ranging from -1.06 to -4.64 (Table 1(Tab. 1)). The compound 4b shows the highest negative dock score of -4.64. All the docked compounds were analyzed for various types of interactions like hydrogen bonding, hydrophobic bonding and Van der Waals interactions with PPAR-γ receptor. The compound 4b binds with PPAR-γ receptor by forming hydrogen bond interactions with amino acid residues HIS370D and ASP517D (Figure 2(Fig. 2)) whereas hydrophobic interactions with amino acid residues LEU500D and SER336D (Figure 3(Fig. 3)) and Van der Waals interactions also. The compound 4b shows similar hydrophobic interactions compared to the standard drug, pioglitazone (Figure 4(Fig. 4)).

### Chemistry

Figure 1(Fig. 1) outlines the synthetic pathway to obtain the title compounds 4a-4h. Ethyl acetoacetate was condensed with thiourea and substituted aldehyde in concentrated hydrochloric acid as a catalyst to get 6-methyl-4-phenyl-2-thioxo-1,2,3,4-tetrahydro-pyrimidine-5-carboxylic acid ethyl esters (1a-1h). The compounds 1a-1h were treated with hydrazine hydrate in ethanol in the presence of the concentrated sulphuric acid as a catalyst to get 6-methyl-2-thioxo-4-phenyl-1,2,3,4-tetrahydro-pyrimidine-5-carboxylic acid hydrazide (2a-2h). The compounds 2a-2h were refluxed with alcoholic potassium hydroxide and para-chloro benzaldehyde to obtain 6-methyl-4-phenyl-2-thioxo-1,2,3,4-tetrahydro-pyrimidine-5-carboxylic acid-N'-(4-formyl-phenyl)-hydrazide (3a-3h). The thiazolidine was synthesized in bulk by refluxing chloroacetic acid and thiourea in an aqueous medium. The compounds 3a-3h in glacial acetic acid were refluxed with thiazolidine and fused sodium acetate to yield the title compounds 4a-4h. The reaction progress and purity of the synthesized compounds were monitored by TLC using silica gel G and by determining its melting point. Structures of the title compounds were confirmed by IR, ^1^H NMR, and mass spectral data.

### Antidiabetic activity

Alloxan-induced beta cell cytotoxicity is a well-established animal model to study antidiabetic activity (Surana et al., 2008[13]). In this study, we observed a decrease in the blood glucose level in both normal and alloxan diabetic rats when treated with the title compounds. The possible mechanism by which the title compounds brings about its hypoglycemic action may be by binding to PPAR-γ receptor as a potent agonist and increase the body's insulin sensitivity (Hauner, 2002[4]). The untreated diabetic rats displayed a significantly higher blood glucose level compared to the control. After treatment with the title compounds, apparently, the title compounds show significant (P<0.001) decrease in the blood glucose level. The compound **4b** shows significant (P<0.001) reduction in the blood glucose level and results are comparable to drug, pioglitazone.

Alloxan treated rats demonstrated substantial weight loss and furthermore influence carbohydrate and lipid metabolism. All the title compounds were found to be effective in restoring the body weight of the animal to a normal value (Table 2(Tab. 2)). 

In a diabetic condition, insulin insufficiency prompts a high serum lipid concentration that causes mobilization of free fatty acids in the peripheral depots. Insulin inhibits the hormone-sensitive lipase in the adipose tissue. Glucagon additionally inhibits the mobilization of free acids from the adipose tissue that results in a decrease in the free fatty acid level in the plasma (Chaudhry et al., 2007[2]). In this study, diabetes-induced animals treated with the title compounds show significant (P<0.001) effect on cholesterol, triglyceride, and LDL level (Table 3(Tab. 3)). The compound **4b** exhibits the highest decrease in cholesterol, triglyceride and LDL level in the reported series, simply as observed in the diabetic rats treated with pioglitazone.

## Conclusion

The present study reveals that potent antidiabetic compounds may be obtained by substituting nonpolar, electron withdrawing substituents at the 4^th^ position of pyrimidine skeleton and hydrogen bond acceptor at the nitrogen of the thiazolidine nucleus, by inhibiting peroxisome proliferator-activated receptor-γ.

## Figures and Tables

**Table 1:**
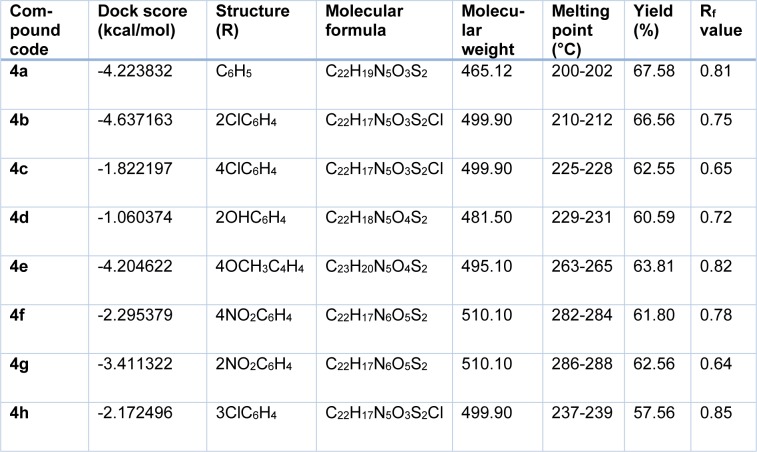
Physicochemical properties of the title compounds

**Table 2:**
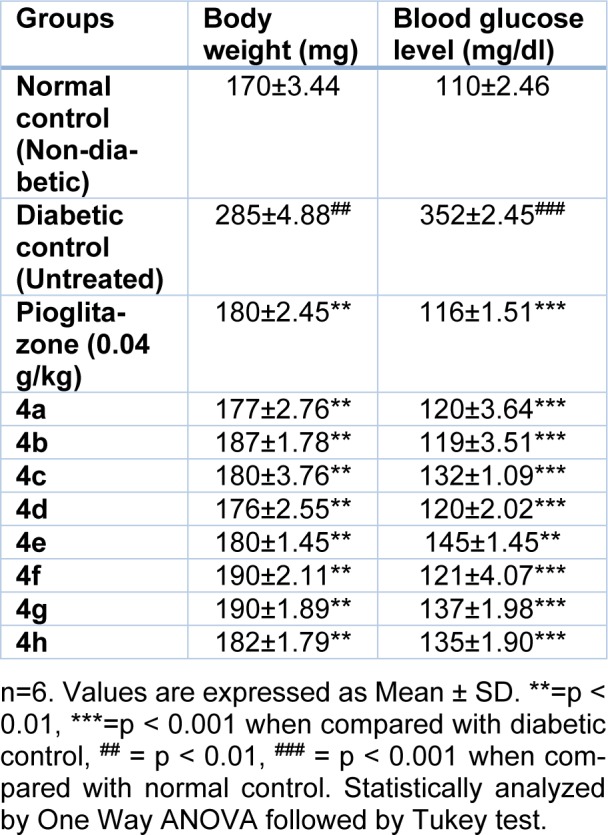
Effect of the title compounds on body weight and blood glucose level

**Table 3:**
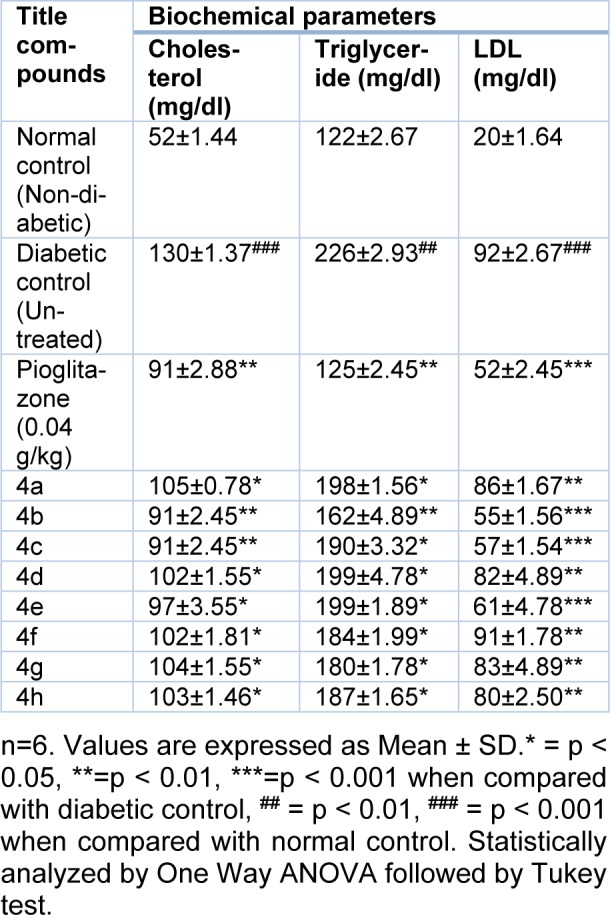
Biochemical parameters of normal and experimental animals on day 8, post treatment
